# Nitrate-Rich Fruit and Vegetable Supplement Reduces Blood Pressure in Normotensive Healthy Young Males without Significantly Altering Flow-Mediated Vasodilation: A Randomized, Double-Blinded, Controlled Trial

**DOI:** 10.1155/2018/1729653

**Published:** 2018-09-16

**Authors:** Karen L. Sweazea, Carol S. Johnston, Brendan Miller, Eric Gumpricht

**Affiliations:** ^1^School of Nutrition and Health Promotion, Arizona State University, Phoenix, AZ, USA; ^2^School of Life Sciences, Arizona State University, Phoenix, AZ, USA; ^3^Isagenix International LLC, Chandler, AZ, USA

## Abstract

Nitric oxide (NO) is a primary vasodilatory factor released from endothelial cells of the peripheral vasculature. NO production is stimulated through enzymatic-dependent mechanisms via NO synthase and from dietary intake of nitrate-containing foods or supplements. We evaluated the efficacy of a nitrate-rich fruit and vegetable liquid supplement (FVS, AMPED NOx, Isagenix International LLC) versus a juice low in nitrates (prune juice, PRU) on circulating nitrates/nitrites as well as cardiovascular parameters in 45 healthy normotensive men (18–40 y). Blood pressure, flow-mediated dilation (FMD), and plasma nitrates and nitrites were measured at baseline and after two weeks of supplementation (2 oz/d). Subjects also completed questionnaires on sleep quality and mood since these measures have been associated with endothelial function. In contrast to PRU, FVS significantly increased plasma nitrates and nitrites (+67%, *p* < 0.001) and decreased diastolic blood pressure (−9%, *p*=0.029) after two weeks. The change in FMD for FVS supplementation versus PRU supplementation was not significant (+2% vs. −9%, respectively, *p*=0.145). Changes in sleep quality or total mood state did not differ between groups after the 2-week study. Thus, the nitrate-rich FVS supplement increased plasma NO and reduced diastolic blood pressure in young normotensive men, but increased plasma NO was not associated with improvements in FMD, mood, or sleep. This trial is registered with ClinicalTrials.gov NCT03486145.

## 1. Introduction

In 2008, Webb et al. postulated that nitrates were one of the bioactive substances in fruits and vegetables responsible for the cardiovascular benefits associated with plant-based diets. These investigators selected beetroot juice, a particularly nitrate-rich food, to test their hypothesis and demonstrated significant reductions in both systolic and diastolic blood pressures several hours after ingestion of beetroot juice (500 ml) [1]. In the past decade, over 30 clinical trials have examined the influence of beetroot juice, specifically, on cardiovascular risk factors. Moreover, a 2013 meta-analysis of 16 eligible randomized crossover trials evaluated the relationship between inorganic nitrate or beetroot juice supplementation and blood pressure verified the strong association of dietary nitrate consumption and blood pressure reduction (mean average reductions: −4.4 mmHg and −1.1 mmHg for systolic and diastolic blood pressure, respectively) [2].

Although receiving less attention than beetroot juice, nitrate-rich green leafy vegetables have shown promising blood pressure-lowering effects and improved endothelial function in healthy adults [3, 4]. Vegetables particularly rich in nitrates include green leafy vegetables such as spinach and lettuce as well as fennel, rocket, radishes, Chinese cabbage, and parsley [3, 5, 6]. In addition to nitrates, green leafy vegetables contain several other nutrients and phytochemicals including vitamins C, E and K, carotenoids (lutein and beta-carotene), flavonols (quercetin and kaempferol), folate, iron, zinc, calcium, and magnesium [3]. According to a recent review, six studies to date have reported inverse associations between consumption of leafy greens (predominantly spinach and lettuce) and cardiovascular disease [3]. It should be noted that seven other studies of green leafy vegetables found no association with cardiovascular disease [3]. Interestingly, the studies that showed benefits on cardiovascular outcomes were conducted mainly in the United States and two of the studies (Nurses' Health Study (NHS) and Health Professionals Follow-up Study (HPFS)) enrolled the largest cohorts (>100,000 female nurses and >50,000 male health professionals, respectively) [3].

Dietary nitrates contribute to nitric oxide (NO) generation in humans (e.g., the nitrate-nitrite-NO pathway), complementing the endothelial cell generation of nitric oxide (NO) from oxidation of L-arginine by nitric oxide synthase [7, 8]. NO promotes endothelium-dependent vasodilation to maintain healthy blood pressure [9]. Dietary nitrates are absorbed in the upper gastrointestinal tract resulting in elevated circulating nitrate concentrations. Approximately 25% of the circulating nitrates are taken up by the salivary glands where they are converted to nitrite by nitrate reductases released from symbiotic bacteria in the oral cavity. When swallowed, some of the nitrite formed in the mouth is protonated in the stomach to form HNO_2_, which decomposes to NO [10, 11]. The remaining nitrate and nitrite are absorbed into the circulation from the upper gastrointestinal tract where they mix with nitrates and nitrites formed endogenously. In the circulation, there are several pathways by which nitrites can also be reduced to NO including conversion by deoxyhaemoglobin and cytochrome P450 [10, 12].

Flow-mediated dilation (FMD) is a noninvasive assessment of vascular endothelial function in humans [13]. There are several reports in the literature examining the impact of beetroot juice ingestion on FMD. A 2013 trial in overweight/obese men demonstrated that acute ingestion of beetroot juice (140 ml containing ∼500 mg nitrates) at mealtime counteracted the adverse effect of the meal on FMD at 2-hours postmeal [14]. Similarly, a 2015 trial in hypertensive men and women that examined the long-term daily consumption of beetroot juice ingestion observed a significant increase in FMD [15].

Consumers are increasingly aware of the health benefits attributed to beetroot as indicated by Google Trends which depicted a 100% increase in the search term “beetroot juice” in the health category over the past five years in the US [16]. *The Grocer* magazine reported that the number of products containing the ingredient beetroot has increased 20% in U.K. markets since 2010 and that statistics from Kantar Worldpanel indicated an 18% increase in sales of fresh beetroot in the U.K. between 2013 and 2015 [17]. Moreover, supplements and juice extracts containing beetroot are popular alternatives to fresh beetroot and are a popular ergogenic aid among competitive athletes [18]. For these reasons, it is important to examine whether these ergogenic aids can improve cardiovascular health. Thus, the primary objective of this study was to examine the short-term effects of a complex novel, two-ounce nitrate-rich fruit and vegetable supplement (FVS) (AMPED NOx, Isagenix International LLC) on plasma nitrates/nitrites and endothelial function in healthy young men. In contrast to other beet juice preparations or beet supplements, FVS sources its nitrates from additional nitrate-rich vegetable extracts beyond beets. Secondarily, because previous studies have demonstrated increased risk for cardiovascular disease, and hence impaired FMD, in individuals with depressed mood, fatigue, or reduced sleep durations [19–21], we also measured mood and sleep quality. Finally, we explored whether self-reported athletic status modified the physiologic responses to the supplement since some research has demonstrated that athletes have improved FMD [22] or alterations in FMD caused by vascular remodeling [23].

## 2. Materials and Methods

### 2.1. Participants

Healthy normotensive men (blood pressure between 100/65 and 140/90 mmHg, [24]) aged 18–40 years were recruited in 2015-2016 from the Phoenix Metropolitan area for this longitudinal parallel arm, randomized, controlled trial through flyers, listservs, and website announcements. Volunteers were prescreened using an online questionnaire, which covered basic demographics including gender, age, height, and weight information as well as athletic status (athlete defined as “actively training for future competitions”). Women were excluded because: (1) the change in progesterone across the menstrual cycle influences FMD [25, 26] and (2) women have more compliant blood vessels less likely influenced by acute dietary changes compared to men in the targeted age range [26]. Additional exclusion criteria were cigarette use within the past year, any food allergies, use of specific medications with vasoactive effects (nitroglycerin, beta-blockers, calcium channel blockers), or unwillingness to consume juice concentrates daily or follow study restrictions. Power calculations indicated that 17 participants per group were necessary to achieve an 80% probability to detect a treatment difference at a 0.05 significance level if the treatment effect for FMD was 2% and assuming the standard deviation (SD) for change is 2% [27]. At the same power and significance level, a much lower sample size was required (*n*=6) to detect a treatment difference (−5 mmHg) for systolic blood pressure assuming the SD for change is 1.4 mmHg [28]. The study was reviewed and approved by the Institutional Review Board at Arizona State University and all participants provided written consent to participate in a study examining the effects of juice supplementation on blood pressure and blood vessel function. Participants were not informed of the contents of each “juice” supplement.

### 2.2. Dietary Supplement (FVS)

The FVS evaluated in this study was provided by Isagenix International LLC (AMPED NOx) and contained ≈240–280 mg (4 mmole) nitrate per two-ounce (60 mL) serving along with ≈51 mg total polyphenols. The FVS contained 7880 mg of a proprietary blend of nitrate-rich extracts from beetroot (*Beta vulgaris*), celery stem and leaf (*Apium graveolens*), and red spinach leaf (*Amaranthus dubius*). Additional ingredients included stevia leaf extract (*Stevia rebaudiana*) and a blend of fruit and vegetable extracts (green tea leaf, red grape, white grape, bilberry, carrot, grapefruit, papaya, pineapple, strawberry, apple, apricot, cherry, orange, broccoli, green cabbage leaf, onion, garlic, black current, asparagus, tomato, olive, and cucumber). The control supplement was prune juice (Sunsweet brand 100% prune juice) (PRU). Prune juice was selected as it has been used as a placebo in prior studies of beetroot juice because it has a similar consistency, caloric, fiber and sugar content, high antioxidant, and phenolic profile, but a low nitrate content [29, 30] (Table 1). The prune juice contained <0.6 mg nitrates and 133 mg total polyphenols per two-ounce serving. All nitrate and total polyphenol analyses were performed by Eurofins Scientific Inc., Des Moines, IA. Both PRU and FVS were provided by Isagenix International LLC in identically sealed, dark plastic bottles labeled either “A” or “B.” Both supplements were similar in appearance, and each bottle contained one serving (two ounces) of the supplement.

### 2.3. Experimental Design

Study investigators and participants were both blinded regarding supplement assignments and the identity of the “A” and “B” supplements were not revealed to study investigators until all data analyses were complete. Participants were stratified by age, height, weight, BMI, and athletic status and randomly assigned to consume daily either FVS or PRU. The subjects were asked to consume one bottle at approximately the same time each morning for 14 consecutive days and to record compliance on a two-week calendar provided on the first study visit. Study compliance was determined as the percent of study days that the supplement was consumed. Participants were additionally asked to report any discomfort or adverse responses to their assigned supplement.

Participants met with investigators on four occasions: screening, baseline (week 0), and study weeks 1 and 2. Participants were instructed not to change their routine dietary or exercise habits during the trial; however, participants were asked to abstain from nonstudy related dietary supplements and to not consume certain vegetables (foods containing >20 mg/100 g nitrates: celery, cress, chervil, lettuce, beets, spinach, rucola, cabbage, endive, fennel, kohlrabi, leeks, parsley, celeriac, dill, turnips, broccoli, carrots, cauliflower, cucumber, pumpkin, and chicory). Since some antimicrobial mouthwashes disrupt the reduction of dietary inorganic nitrates to nitrites by commensal bacteria in the mouth [31], participants were also asked to refrain from using antiseptic mouthwashes for 21 consecutive days starting one week prior to initiation of the 2-week intervention. Prior to each testing visit (baseline, week 1 and 2), participants were instructed to fast overnight (no food or drink with the exception of water for at least 10 h). In addition, participants were asked to abstain from caffeine for 24 hours and exercise for 48 hours prior to testing. Participants were asked to arrive at the laboratory between 07:00–10:00 am for each testing visit and were seated in a semisupine position in a dimly lit room for at least 20 minutes prior to measuring blood pressure using a Medline Automatic Digital Blood Pressure monitor (Medline Industries Inc., Mundelein, IL). Blood pressure was recorded for three consecutive measurements with the 2nd and 3rd measurements averaged for the final value. Next, flow-mediated vasodilation in the brachial artery was measured by a trained sonographer as described below. Following the FMD measurement, fasting blood samples were collected using vacutainers coated with EDTA and centrifuged at 3,000 rpm for 15 min at 2–4°C. Plasma was aliquoted into microcentrifuge tubes for the analysis of nitrates and nitrites and stored at −80°C until analyses. Body mass, % body fat, and body mass index (BMI, in kg/m^2^) were measured at each study visit using a calibrated Tanita bioelectrical impedance analyzer (model TBF-300A, Tanita Corporation, Tokyo, Japan). Waist circumference was measured in cm at the midpoint between the lowest palpable rib and the iliac crest using a flexible tension tape according to guidelines from the World Health Organization [32]. Height was measured using a wall-mounted stadiometer. All measurements were performed by the same study investigator. Physical activity was assessed using a validated questionnaire that quantifies physical activity in terms of metabolic equivalents (METS) [24].

### 2.4. Flow-Mediated Vasodilation (FMD) and Blood Analyses

FMD was measured according to the criteria described by the Brachial Artery Reactivity Task Force [33, 34]. After measuring baseline blood pressure, the sonographer measured the baseline (preocclusion) diameter of the left brachial artery for 60 seconds using a high-resolution ultrasound machine (t3000; Terason Ultrasound, Burlington, MA) with a 10 MHz multifrequency linear array probe. A blood pressure cuff was positioned distal to the ultrasound probe and was inflated to a pressure of ≥40 mmHg above systolic arterial pressure for 5 minutes. Images were recorded during the final 60 seconds of the occlusion to measure the minimum occlusion diameter after which the cuff was rapidly deflated to elicit a reactive hyperemic stimulus that is considered predominantly endothelium-mediated and NO dependent [13]. Images were recorded and analyzed continuously for the following 5 minutes and analyzed to determine the % peak dilation using validated brachial artery edge-detection software [35, 36] by the sonographer who was blinded as to the treatment groups. The position of the probe was recorded for each participant to ensure that it was placed at the same location for each testing. Intraclass correlation coefficients for this technique in our laboratory for baseline and peak diameter are 0.994 and 0.995, respectively (Cronbach alpha = 0.976).

As the half-life of nitrite is only 110 s, and levels are often below detectable levels using conventional analyses, studies often report the total nitrate and nitrite concentrations in plasma as an estimate of NO bioavailability [37]. Plasma total nitrates and nitrites were measured using a commercially available colorimetric assay kit (Cat. no. 78001; Cayman Chemical, Ann Arbor, MI). Plasma was prefiltered through 30 kDa molecular weight cutoff ultrafilters (Millipore, Billerica, MA) according to the assay protocol as large plasma proteins may interfere with analyte determination. The assay uses nitrate reductase to convert sample nitrates to nitrites, which are then complexed to Griess reagent forming a purple azo compound detected spectrophotometrically. The intraassay %CV for total nitrates and nitrites was <10%.

### 2.5. Mood Assessment and Sleep Quality

At each testing visit, participants completed a Profile of Mood States (POMS) questionnaire, a validated measure for assessing six mood states: tension, depression, anger, vigor, fatigue, and confusion [38]. Participants were told to report their feelings during the past week, and a total mood score was calculated (the sum of the tension, depression, anger, fatigue, and confusion scores minus the fatigue score). Based on an age, gender, and race-stratified sample of healthy adults representative of the U.S. population (*n*=400), a normative reference for the total mood score is 14 [39]. Higher POMS scores indicate greater affect. To assess sleep quality, participants completed the Pittsburgh Sleep Quality Inventory (PSQI), a validated measure composed of seven component scores (sleep quality, sleep latency, sleep duration, habitual sleep efficiency, sleep disturbances, use of sleeping medications, and daytime dysfunction) that are summed to produce a global PSQI score [40]. In their evaluation of PSQI, Buysse et al. reported a mean global score for healthy adults of 2.7 and suggested that scores >5 indicate a degree of sleep disturbance [40].

### 2.6. Statistical Analyses

Data are presented as the means ± standard deviation (SD). The data were analyzed using SPSS version 23 (IBM, 2015, Chicago, IL). Differences in baseline characteristics were examined by independent *T*-test, and the two-week change in outcome variables by group were examined using univariate analyses controlling for baseline values. Data were checked for normality and transformed when necessary. Age and physical activity level were not related to outcome variables; however, since markers of adiposity were related to the outcome variables, body mass was entered as a covariate in all analyses. Alpha was set at 0.05.

## 3. Results

**Figure 1:**
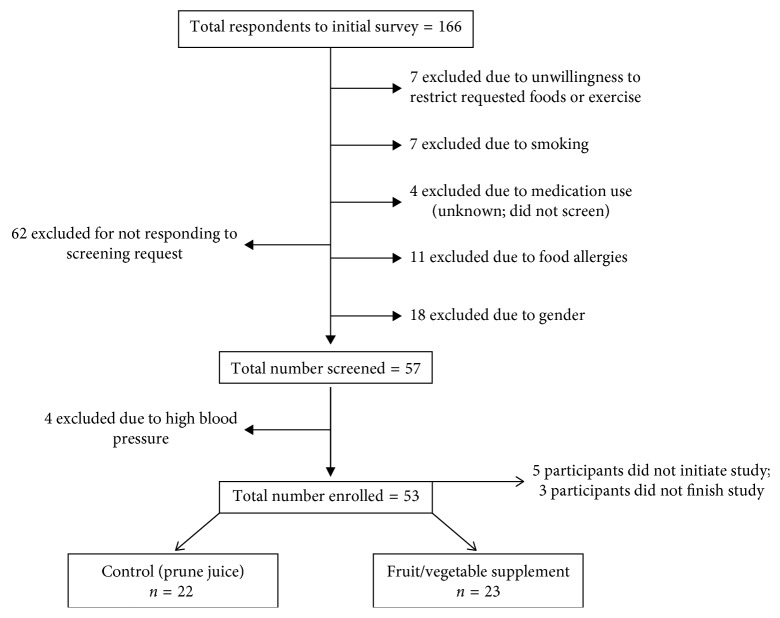
Consort diagram.

Of the 166 volunteers who were prescreened for eligibility, 57 participants met the inclusion criteria and were enrolled in the study; however, three participants were withdrawn prior to the start of the study based on exclusion criteria and an additional six participants did not initiate the study (Figure 1). While 48 participants initiated the study, three participants did not complete the two-week intervention due to personal conflicts (two in the PRU group and one in the FVS group; Figure 1). Data are reported for the 45 participants who completed the trial (Figure 1). Study compliance (percent of study days that juice was consumed) was excellent for both groups: 98% and 99% for FVS and PRU, respectively. Baseline characteristics did not differ by group (Table 2); furthermore, athletic status did not relate to any of the outcome measures at baseline and was not associated with changes in any of the outcome variables after the 2-week intervention.

**Figure 2:**
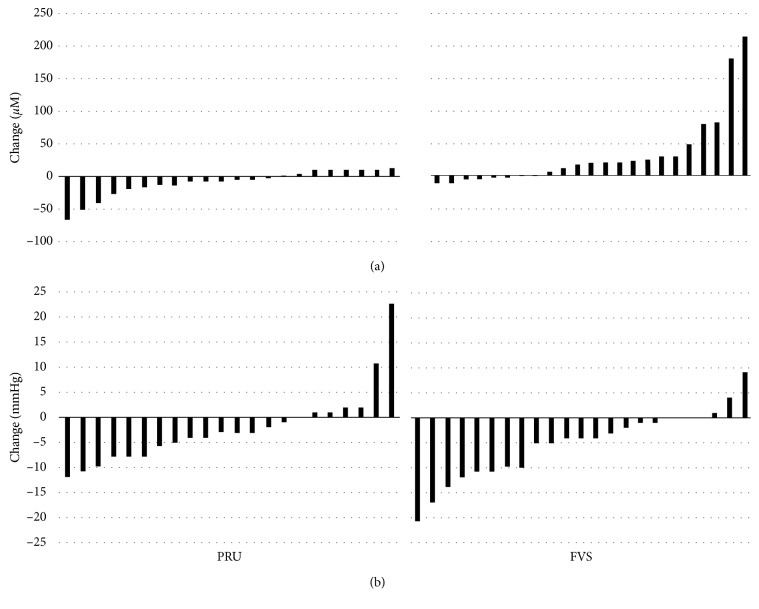
Two-week change in (a) plasma nitrates/nitrites and (b) diastolic blood pressure in individual participants consuming two ounces of a fruit and vegetable blend (FVS; *n*=23) or prune juice (PRU; *n*=22).

Diastolic, but not systolic, blood pressure fell significantly for participants in the FVS group over the course of the 2-week study in comparison to the PRU group (−5.3 ± 7.0 and −2.2 ± 7.7 mmHg respectively, *p*=0.028) (Table 3). The change in FMD from baseline to 2 weeks did not differ between groups (+0.1 ± 2.8% and −0.5 ± 4.2% for the FVS and PRU groups, respectively; *p*=0.145). By week one, plasma nitrates and nitrites rose markedly in the FVS group in comparison to the PRU group and levels remained elevated at week 2 (34.1 ± 57.6 and −9.7 ± 21.0 *µ*M, respectively, *p*=0.001). Changes in diastolic blood pressure and plasma nitrates and nitrites for individual participants are shown in Figure 2. Total mood state and sleep quality did not vary between treatment groups over the course of the study (Table 3).

## 4. Discussion

The purpose of the current study was to evaluate the effect of short-term supplementation of a novel nitrate-rich FVS supplement on plasma nitrates/nitrites and cardiovascular parameters in healthy young men. These data demonstrate that daily ingestion of a two-ounce FVS shot for two weeks significantly increased total plasma nitrates/nitrites and reduced diastolic blood pressure in comparison to a low-nitrate control juice. However, a recent meta-analysis of 16 randomized controlled crossover trials with beet juice reported reductions in both systolic and diastolic blood pressures, but noted a greater effect of beet juice on systolic blood pressure (−4.4 versus −1.1 mmHg for systolic and diastolic, respectively) [2]. It is possible that a direct comparison between effects of beet juice *per se* and the FVS evaluated in the current study may not be equivalent since the nitrate-rich sources in FVS include not only beet but also celery and red spinach extracts. In this context, Bondonno et al. [41] in a randomized, controlled, crossover trial in both healthy men and women reported that 200 g spinach, containing 3 mmoles nitrate, significantly increased plasma nitrates/nitrites, enhanced FMD, and lowered systolic (but not diastolic) blood pressure. Although not entirely consistent with previous reports, the hypotensive effect of the FVS provides additional support for the inclusion of nitrate-rich foods or supplements as potentially safe and effective nutritional strategies for regulating normal blood pressure.

Blood pressure levels are a strong predictor of both vascular and nonvascular diseases, and their predictive power for vascular disease is stronger than that for blood cholesterol concentration or cigarette use [42]. Hence, the value of identifying strategies to lower blood pressure is clear. Recent meta-analyses support the efficacy of low-calorie diets, low-sodium diets, low-sodium/high potassium diets, and vegetarian diets for reducing blood pressure, with mean reductions in systolic and diastolic blood pressures in the ranges of 5–8 mmHg and 2–4 mmHg, respectively [43–45]. Although chlorogenic acid, a component of prune juice, has been shown to reduce blood pressure according to a recent meta-analysis [46], no such blood pressure-lowering effects were observed in subjects consuming PRU in the present study. In the current study, diastolic blood pressure fell 5 mmHg for participants in the FVS group, a change that has been associated with a 20–30% reduction in risk for coronary heart disease [47, 48]. The daily consumption of a dietary supplement, e.g., 2 ounces of a nitrate-rich fruit and vegetable extract as used in this trial, may be easier to implement and have higher adherence rates than the more comprehensive diet change required for reducing calories or sodium in the diet.

Interestingly, the observed hypotensive effect of the FVS did not correspond with an effect on FMD, a commonly used, noninvasive assessment of vascular endothelial function. This discordance has previously been noted in other human intervention trials with beet juice. For example, while some investigations noted a lack of influence of dietary nitrates on FMD [28, 49], others found a greater FMD in response to dietary nitrate consumption [1, 50]. Of particular relevance, however, is that enhanced FMD by beet juice is typically noted within 3 hrs of nitrate consumption [1]. Because of the purpose and design of the current study, FMD assessments were performed ≈24 hours after the last consumed dose of FVS to measure chronic effects of the supplement. Therefore, it is possible that acute changes in FMD may have occurred following immediate supplementation but these effects resolved by the following days' analysis. Moreover, the assessments evaluated FMD in healthy subjects without additional dietary treatments. Therefore, it is possible that any beneficial effect of the supplement may have required experimentally-induced endothelial dysfunction to demonstrate an effect such as that observed postprandially after a high-fat meal [14].

The average baseline FMD values for participants were below those reported in recent reviews [22, 23]; hence, a ceiling effect was not likely in this study. Several possibilities may have contributed to the lack of influence of FVS on FMD. First, the level of nitrates in the extract (≈240–280 mg/4 mmole per two-ounce shot) may not have been sufficient to influence FMD as previous studies have noted a strong association between the dose of nitrates and magnitude of nitrate-facilitated improvements toward endothelial dilation. For example, Lara et al. [51] recently demonstrated that trials utilizing nitrate dosages above 600 mg reported the most substantial change in endothelial function. Despite the acceptance of FMD as an outcome marker to assess the efficacy of various treatment modalities, the 2010 policy guideline of the American College of Cardiology Foundation/American Heart Association concluded that this assessment tool should not be used for cardiovascular risk assessment in asymptomatic adults [52], in part due to the extreme interindividual variability reported among published values [53].

Although highly significant, the elevation of plasma nitrates/nitrites by the FVS shot was less than that observed in most studies. This was most likely explained the by lower concentration of dietary nitrates provided by FVS compared with dosages chosen in previous studies. Furthermore, participants were instructed to restrict dietary nitrate consumption to only that provided in the FVS. Thus, the hypotensive effect of the FVS towards diastolic blood pressure was most likely due to the supplement's nitrate content, although it is possible that other biologically active components may have contributed to or altered the blood pressure-lowering effects of FVS. For example, a study comparing blended apples enriched with additional apple skins (rich in flavonoids) and spinach (rich in nitrates) found both increased plasma NO and FMD resulting in lower systolic blood pressure compared to a control [41]. Flavonoids are thought to increase NO bioavailability through increased eNOS activity and thus production of NO as well as through inhibition of endothelial NADPH oxidase [41]. In fact, increases in FMD were greater with the consumption of apples as compared to spinach, even though plasma NO was increased more in response to spinach supplementation [41]. Surprisingly, when apples and spinach were consumed together, there were no additive effects on blood pressure. Bondonno et al. [41], attributed the lack of an additive vascular response to enhanced reduction of nitrites to NO in the gut, resulting in less nitrite absorption into the circulation for vascular reduction to NO [41]. Polyphenols can also improve conversion of dietary nitrates to NO in the gut [5]. Foods rich in flavonoids include tea, citrus, berries, red wine, apples, and legumes. Thus, it is possible that a similar reduction of nitrite in the gut by flavonoids or polyphenols in the FVS may have limited the effects of the supplement on systolic blood pressure and FMD.

Prune juice was chosen as a control since this beverage has a very low nitrate content, yet similar caloric and sugar content as FVS, and has functioned as a placebo in a previous study [29]. Although total polyphenols were higher in the prune juice compared to the FVS, and thus not likely a factor in the FVS-induced reduction in diastolic blood pressure, the contribution of total polyphenols in these supplements was much below the estimated dietary intake of total polyphenols (≈1000 mg/day) [54, 55]. Fruit and vegetables are a rich source of potassium, and the possible impact of potassium intake on reductions in diastolic blood pressure cannot be ruled out since the potassium level in FVS is not known. The potassium content of the prune juice is 177 mg/2 oz. However, diastolic blood pressure in the FVS participants at the end of the study was significantly related to blood nitrate concentrations (*r*=−0.455, *p*=0.029) suggesting that the hypotensive property of the FVS noted herein was related to its nitrate content.

There are additional limitations to the study results. First, healthy male subjects were specifically recruited due to their greater risk of developing cardiovascular disease, and women were excluded to avoid potential menstrual cycle-influenced fluctuations in progesterone known to influence vascular reactivity. Therefore, it is unknown whether the supplement would have provided a similar hypotensive response in women. In other studies, a gender-specific response to beet juice supplementation has been reported to induce antiplatelet effects in males, but not females, consuming beet juice [56], perhaps attributed to gender differences in nitrate/nitrite homeostasis and metabolism [57]. Another limitation is the use of a parallel arm study design which may have limited the power of the study and contributed to the number of confounding factors such as dietary intake.

While any physiological pathway that may link endothelial function with either sleep or mood status is uncharacterized, several researchers have reported strong inverse correlations between FMD and impaired mood or sleep states [58–60]. Therefore, we assessed influence of a potential nitrate-induced enhancement of FMD toward these parameters of overall well-being. However, mood and sleep scores were not altered by daily ingestion of FVS despite a significant elevation in plasma nitrates and nitrites. As discussed above, the nitrate dose of the supplement may have been insufficient to change endothelial function as measured by FMD; hence, any influence of FMD on mood and sleep could not be demonstrated.

## 5. Conclusions

Daily consumption of a two-ounce FVS raised blood nitrates/nitrites and resulted in a hypotensive effect in normal healthy males. This increase in plasma nitrates/nitrites was not associated with changes in FMD, mood or sleep. While the implications of these results will require further evaluation, this study adds to a growing body of evidence suggesting that even small amounts of dietary nitrates may be an effective nutritional strategy to regulate healthy diastolic blood pressure and provide cardioprotection.

## Figures and Tables

**Table 1:** Nutrition facts for the study supplements^1^.

Nutrient	FVS	PRU
Calories	30	45
Total carbohydrates	6 g	11 g
Dietary fiber	1 g	<1 g
Sugars	2 g	6 g
Protein	1 g	<1 g
Dietary nitrates	240–280 mg	<0.6 mg
Total polyphenols	51 mg	133 mg

^1^FVS: fruit and vegetable extract; PRU: prune juice. FVS data from manufacture; PRU data from http://sunsweet.com.

**Table 2:** Baseline characteristics of study Participants^1^.

Participant characteristics	FVS	PRU	*p* value
	(*n*=23)	(*n*=22)	
Age, y	24.0 ± 4.3	24.4 ± 34.4	0.695
Body mass index, kg/m^2^	25.6 ± 3.5	25.2 ± 3.5	0.645
Body mass, kg	81.9 ± 12.5	78.2 ± 13.6	0.305
Waist circumference, cm	33.1 ± 3.8	33.0 ± 3.8	0.913
METs, kcal·kg^−1^·wk^−1^	76.7 ± 46.8	64.9 ± 32.9	0.081

^1^Data are the mean ± SD. *p* value represents independent *t*-test; data were transformed to normalize. FVS: fruit and vegetable supplement; PRU: prune juice; METs: Metabolic Equivalent for Task.

**Table 3:** Outcome measures at baseline and study weeks 1 and 2 in participants consuming two ounces FVS (*n*=23) or PRU (*n*=22) daily for two weeks^1^.

Variable	Baseline	Week 1	Week 2	2-week change	*p(Effect size)*
Systolic BP, mmHg					
FVS	116.2 ± 6.5	116.1 ± 9.1	112.1 ± 8.3	−4.1 ± 7.5	0.370
PRU	117.7 ± 10.1	115.2 ± 7.7	115.7 ± 7.1	−2.0 ± 8.5	*(0.020)*
Diastolic BP, mmHg					
FVS	61.7 ± 7.7	61.7 ± 7.7	57.4 ± 6.2	−5.3 ± 7.0	**0.028**
PRU	62.3 ± 8.2	62.3 ± 8.2	62.9 ± 8.0	−2.2 ± 7.7	*(0.112)*
FMD, %peak dilation					
FVS	6.3 ± 2.7	6.5 ± 2.9	6.4 ± 2.6	0.1 ± 2.8	0.145
PRU	5.7 ± 2.6	5.5 ± 2.4	5.2 ± 3.0	−0.5 ± 4.2	*(0.051)*
Plasma nitrates and nitrites, *µ*M					
FVS	27.4 ± 23.5	63.3 ± 67.7	61.5 ± 58.5	34.1 ± 57.6	**0.001**
PRU	38.7 ± 37.0	31.8 ± 30.4	29.1 ± 25.5	−9.7 ± 21.0	*(0.244)*
POMS					
FVS	13.5 ± 19.0	10.7 ± 20.8	9.2 ± 17.3	−4.0 ± 19.1	0.369
PRU	10.1 ± 22.7	1.5 ± 14.7	2.9 ± 21.7	−7.3 ± 9.9	*(0.020)*
PSQI					
FVS	4.3 ± 2.4	4.5 ± 2.4	4.2 ± 2.6	−0.1 ± 1.7	0.970
PRU	4.1 ± 2.3	3.8 ± 2.1	4.0 ± 2.7	−0.2 ± 2.0	*(0.000)*

^1^Data are mean ± SD; FVS: fruit and vegetable extract, PRU: prune juice. Nonnormal data were transformed prior to analyses (blood pressure; plasma nitrates/nitrites). There were no differences between groups at baseline. *p* value represents univariate analyses for change data controlling for baseline value and body weight.

## Data Availability

The data used to support the findings of this study are available from the corresponding author upon request.
